# Continuous or dicrete? Attractor dynamics and spatial representations in a model of the hippocampal network

**DOI:** 10.1186/1471-2202-14-S1-P397

**Published:** 2013-07-08

**Authors:** Federico Stella, Remi Monasson, Alessandro Treves

**Affiliations:** 1 Cognitive Neuroscience Sector, Sissa, Trieste, Italy; 2 Laboratorie de Physique Theorique, ENS, Paris, France; 3 Embassy of Italy, Science Office, Tel Aviv, Israel

## 

There is now strong evidence of the presence of a vast integrated neural system, which involves the hippocampus and several parahippocampal regions, dedicated to spatial navigation and memory. 'Place cells' and 'Grid cells' have been identified as neural correlates of the ability to remember an environment and to successfully move through it.

The storage of a spatial map in the hippocampal circuits poses important computational issues. It asks for an extension of the classical notion of attractor, successfully used to model the storage of discrete memory items, like episodic memories, to a new entity that captures the continuous nature of space.

In a continuous attractor activity smoothly changes with the position of the animal in the environment, so that each point in space is uniquely represented in the hippocampus. This situation corresponds to the presence of an infinite number of distinct stable configurations of the system, each for a different point of the environment.

It is not known how much this idealized notion corresponds to the actual structure of the maps stored in the hippocampus, and how well attractor dynamics can approximate a continuous representation.

We address these questions, within a simplified mathematical network model. The model network simulates the storage in CA3 of a spatial representation, its retrieval and its transfer to CA1. Through both analytical calculations and computer simulations we evaluate the ability of the network to represent different points in space and we study the properties of the attractor landscape when different kinds of disorder (connections dilution, heterogeneous place field distribution, multiple maps) are introduced in the system. Moreover we investigate the possible role of CA1 in the realization of a continuous attractor.

We find that even networks of considerable size can only approximate the idealized notion of a 2D quasi-continuous dynamical attractor, but that the presence of CA1 generates a 'smoother' representation of space.

